# *Mycoplasma hominis* and *Ureaplasma urealyticum* infections after knee arthroplasty: A case report

**DOI:** 10.1097/MD.0000000000031202

**Published:** 2022-11-11

**Authors:** Jingjing Luo, Xinan Wu, Xue Gang, Nana Zhang, Feifei Wang, Chengting Rong

**Affiliations:** a Department of Clinical Pharmacy, Hefei BOE Hospital, An’hui, Hefei, P.R. China; b Department of Orthopaedics, Hefei BOE Hospital, An’hui, Hefei, P.R. China.

**Keywords:** case report, joint infection, *Mycoplasma hominis*, postoperative infection, *Ureaplasma urealyticum*

## Abstract

Artificial joint infection caused by *Mycoplasma hominis* and *Ureaplasma urealyticum* is rare and has not been reported.

**Patients concerns:**

A 59-year-old man underwent left total knee arthroplasty for 1 year of pain in the left knee joint. The indwelling urinary catheter was removed after 48 hour of the surgery. On day 8 after the surgery, the patient had fever, increased skin temperature, swelling and redness around the surgical site, and floating patella test (+). According to experience, Vancomycin, Ciprofloxacin and Linezolid were administrated. Evident decrease in C-reactive protein was observed after Linezolid administration, while there was no significant improvement in clinical symptoms. Microbiome sequencing was performed, resulting in diagnosis of positive *M hominis* and *U urealyticum*. The patient was then treated with Doxycycline in the following 3 months. During the 11-month outpatient follow-up, there was no evidence of recurrence of infection.

**Diagnosis:**

Microbiome sequencing was performed, resulting in diagnosis of positive *M hominis* and Ureaplasma urealyticum.

**Interventions:**

The patient recovered following with Doxycycline in the following 3 months.

**Outcomes:**

During the 11-month outpatient follow-up, there was no evidence of recurrence of infection.

**Lessons:**

*M hominis* and *U urealyticum* are common pathogens of the urinary system infections but they are rare in osteoarticular infections. In cases of fever, swelling and heat pain around the surgical site, joint fluid, negative blood culture and being irresponsive to anti-bacterial agents against the cell wall, special bacteria-related infection should be highly suspected.

## 1. Introduction

Arthroplasty has been increasingly popular in patients with osteoarthritis or arthritis given its excellent efficacy in alleviating pain and improving joint mobility. Prosthetic joint infection is rare. Negative cultures, may result from pre-culture antibiotics administration, or the condition that the routine treatment fails to identify the responsible pathogens.

*Mycoplasma hominis* and *Ureaplasma urealyticum* tend to be adherent to the mucosal epithelial cells of the urogenital tract, leading to urinary system, and gynaecological infections. In addition, they can also cause infections outside the urogenital system, such as sepsis, central nervous system infection, respiratory infection, joint infection, and wound infection, which are mainly due to the mucosal injuries (such as mechanical operation, surgery and trauma) and immune dysfunction.^[1]^ Here, we report a case who suffered from infections caused by *M hominis* and *U urealyticum* after knee arthroplasty.

## 2. Case presentation

A 59-year-old man was admitted to Hefei BOE Hospital on May 10, 2021 for 1 year of pain/discomfort in the left knee joint. Admission physical examination revealed body temperature = 36.6 °C, heart rate = 84 beats/minute, respiratory = 20 breaths/minute, and blood pressure = 110/75 mm Hg (1 mm Hg = 0.133 kPa). The patient was conscious with a good spirit, continent, and had a normal diet and body weight. Both lower limbs were basically equal in length. The right lower-limb muscle atrophy was evident with the muscle strength decreased to grade III, while the sensory function of the limb was normal and the end vascular actions were good. Mild edema was observed in the left knee joint, but the skin temperature was normal and there was no tenderness. In addition, the left lower limb was slightly limited in movement with a range of around 5 to 100° and a sensation of bone friction. The sensory function of the left lower limb was normal and the end vascular actions were good. The patient was initially diagnosed with unilateral (left) knee osteoarthritis, which was confirmed after admission.

**Figure 1.**
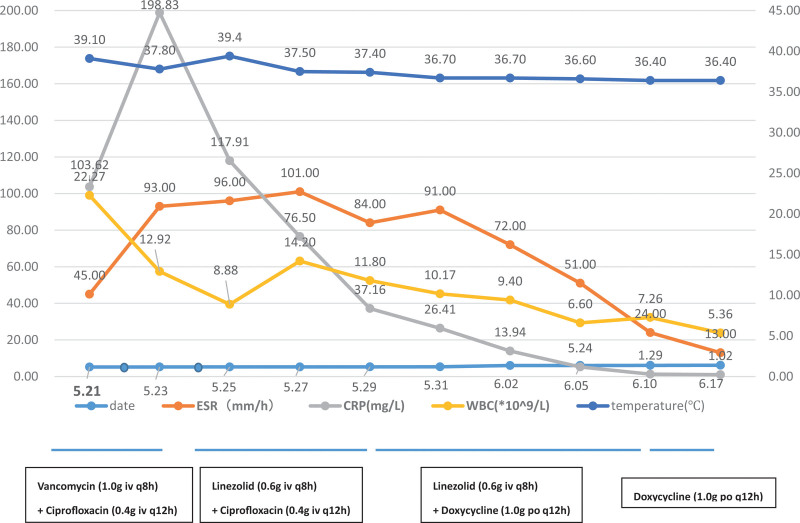
Relationship between the change of inflammatory markers (WBC, ESR, and CRP) and the use of antibiotics during hospitalization. CRP = C-reactive protein, ESR = erythrocyte sedimentation rate, WBC = white blood cells.

The patient was in good general condition and had stable vital signs after admission. There was no evidence of abnormality in preoperative examinations. On May 13, the patient underwent left total knee arthroplasty, with prophylactic Cefuroxime (1.5 g, ivgtt, q8h) administrated for 48 hour. In the meantime, a urinary catheter was indwelled during the procedure and then removed 48 hour after the operation. Other treatments included anticoagulation using Nadroparin Calcium (0.4 mL, iv, qd), analgesia with Parecoxib (40 mg, ivgtt, bid) plus Oxycodone Sustained-Release Tablet (10 mg, po, q12h) and cold therapy as an adjuvant to relieve the swelling and pain. On May 21, the patient had a fever and reached the highest body temperature of 39.1 °C (Fig. 1). Laboratory examination revealed significantly increased white blood cells (WBC), ultrasensitive C-peptides and erythrocyte sedimentation rate (ESR) (Fig. 1). There was obvious redness around the surgical site, and positive tenderness and floating patella test were obtained. Movement of the knee joint was good, and the sensory function and end vascular actions of the lower limbs were good as well. Magnetic resonance imaging (MRI) showed hydrops in the joint cavity with swelling of the surrounding soft tissues. On May 21 and 24, knee joint puncture was performed on the surgical site. The puncture fluid was in dark red and negative for bacteria and acid-fast bacilli by blood culture and puncture fluid smear, respectively. In the meantime, negative results were also shown in blood culture and drug sensitivity test under aerobic or anaerobic conditions. On May 21, Vancomycin (1.0 g, ivgtt, q8h) and Ciprofloxacin (0.4 g, ivgtt, q12h) were given by experience (Fig. 1). On May 24, the blood drug concentration of Vancomycin was 10.44 ug/mL. The body temperature failed to fall significantly and the highest reached 39 °C. There was no improvement in the surgical site but progressive aggravation of the redness and swelling. On May 25, Vancomycin was replaced by Linezolid (0.6 g, ivgtt, q12h)^[2]^ and Ciprofloxacin was continued. PIseq™DNA + RNA was then performed. The patient had slight decline in body temperature in the following 5 days but the highest still reached 37.6 °C. Reductions in inflammatory indicators, especially C-reactive protein (CRP), were also observed (Fig. 1), while the redness and heat pain in the surgical site persisted. On May 30, PIseq™DNA + RNA result revealed growth of *M hominis* and *U urealyticum*. Ciprofloxacin was then replaced by Doxycycline with a loading dose of 0.2 g followed by 0.1 g, po, q12h. The body temperature recovered to normal on the next day, and the regional rednessand heat pain got relieved significantly. On June 15, Linezolid was discontinued. On June 17, the clinical symptoms and inflammatory indicators of the patient gradually recovered to normal. The patient was then discharged and asked to take Doxycycline for 2 more months. During the 11-month outpatient follow-up, there was neither significant adverse drug reaction nor symptomor signs of reinfection.

## 3. Discussion

Mycoplasma is a general term of any microorganisms of the Mollicutes. It has been established that the *Mycoplasma* species are the smallest self-replicating organisms. Currently, there have been four *Mycoplasma* species identified in human, including *Mycoplasma pneumoniae*, *M hominis*, *Mycoplasma genitalium* and *U urealyticum*.^[3]^ *M hominis* and *U urealyticum* with no cell wall can survive independently, which enables them to be naturally resistant to glycopeptides, such as Penicillin, Cephalosporin, β-lactams, and Vancomycin. In addition, they cannot be detected by Gram staining. To know better about the characteristics of infections with both *M hominis* and *U urealyticum* after joint arthroplasty, authors of the study searched CNKI, PubMed, etc, and found that there was no relevant report as of July 2021 domestically and abroad. Most reported cases were infected with single pathogens. For example, Lili Xiang et al^[4]^ and Luttrell LM et al^[5]^ analyzed a total of 7 cases who were infected with *M hominis* after arthroplasty, including 4 cases receiving knee arthroplasty, 2 cases undergoing hip arthroplasty and 1 case experiencing shoulder arthroplasty. In addition, Farrell et al,^[6]^ MacKenzie et al^[7]^ and Sköldenberg et al^[8]^ reported 3 cases of *U urealyticum* infection after arthroplasty. In most cases of infection, the patients will present fever, increase in WBC, ultrasensitive C-peptide and ESR, redness, heat pain, and exudation in the surgical site, and they are generally irresponsive to β-lactams. The specific source of infection remains to be clarified. One possible source is the invasive operations to the urinary tract performed before arthroplasty in most patients. Additionally, the reproductive system infection and the hematogenous dissemination to the surgical site are also possible sources.^[9]^ The case reported here had redness and heat pain in the surgical site after knee joint arthroplasty, accompanied by significant elevation of WBC, ESR, and ultrasensitive C-peptides most obvious. In the meantime, the patient was free of any β-lactams and irresponsive to Vancomycin and Ciprofloxacin. No Genitourinary infection, a possible primary source of septic arthritis reported in the medical literature, was ruled out in our case by urine culture. However, invasive operation of the urinary system was considered as a possible source of knee joint infection after surgery as the patient carried a urinary catheter during the procedure.

Microbiological tests for *M hominis* and *Ureaplasma*, such as Gram staining and early routine culture, generally are negative or insensitive, given requirements of specific operations and culture medium.^[10]^ Besides, such kinds of pathogens are not covered in most hospitals where microbiological tests are performed for infections outside the urogenital system only. It is believed that microbiome gene sequencing can be a viable option upon negative cultures.^[10]^ Research found that nucleic acid tests, such as PCR, are time-saving (within one day) and commonly highly sensitive to microbiomes than cultures.^[11]^ However, they are not available in a large number of hospitals. In the present case, negative bacterial and fungi cultures were obtained by two times of blood culture and joint puncture fluid smear. Microbiome gene sequencing was then performed and infections caused by *M hominis* and *U urealyticum* were confirmed.

It has been established that *M hominis* and *Ureaplasma* are generally sensitive to Tetracyclines, Macrolides and Quinolones. Recent studies have revealed that both of the two pathogens are highly resistant to Quinolones but they still have a high sensitivity (>70%) to Doxycycline and Minocycline.^[12]^ Here, the patient had a proper blood drug concentration of Vancomycin, but no evident improvement was observed. We reasoned that this might be due to the development of drug resistance or the insufficiency of regional drug concentration. Referring to the literature, we noted that Vancomycin concentration in bone tissue is low (7–13 ug/mL) but Linezolid concentration can be up to 60 ug/mL. Vancomycin was then replaced by Linezolid, contributing to reductions of CRP and body temperature. However, the patient was persistently hypothermic and the surgical site still showed redness and heat pain. Certain clinical efficacy was achieved. Kenny et al^[13]^ reported that Linezolid was active on *M hominis* but inactive on *U urealyticum* when its blood drug concentration reached 8.0 ug/mL (MIC50). Based on this study, we speculated that the patient here responded well to Linezolid, probably because Linezolid was only active on *M hominis* at that time. Nevertheless, whether the activity of Linezolid against *M hominis* is dependent on its blood concentration could not be determined. Fang et al^[14]^ suggested that Linezolid was not recommended for treatment of *M hominis* infections, as they found that Linezolid with a blood drug concentration of over 8.0 ug/ml was associated with a high incidence of thrombocytopenia in Chinese population, and the range between 2 to 7 ug/mL seems to be safe and effective.

Here, drug sensitivity test was not performed. The patient was not effectively treated with Ciprofloxacin until replacement by Linezolid, considering resistance to Quinolones. According to *Harrison’s ^TM^ Infectious Diseases*, Doxycycline, which is highly sensitive to *M hominis* and *U urealyticum*, was administrated in this patient. On the next day, the patient had normal body temperature and showed significant relief of symptoms of surgical site infection with reduced inflammatory indicators. Furthermore, Doxycycline was continued for 2 more weeks and the clinical symptoms and indicators of infection recovered to normal. Linezolid was discontinued on June 15. The patient was discharged on June 17 and asked to take Doxycycline for 2 more months.

To conclude, it is rare to have *M hominis* or *U urealyticum* infection after joint arthroplasty, and there was no related case reported before suffering from infections caused by both pathogens. The present case suggested that, upon negative bacterial/fungal test and failure of treatment with Vancomycin or β-lactams, *M hominis* or *U urealyticum* infection should be considered. In addition, Tetracyclines (such as Doxycycline) with high sensitivity to these kinds of pathogens can be administrated if drug sensitivity test result is not available. Furthermore, microbiome gene sequencing can be an option in cases with negative culture results. It is notable that Linezolid is active on *M hominis* but inactive on *U urealyticum*. It is not recommended for treatment as there is a high risk of thrombocytopenia when the blood drug concentration is over 8.0 ug/mL.

## Author contributions

**Conceptualization:** Jingjing Luo.

**Data curation:** Jingjing Luo.

**Formal analysis:** Jingjing Luo, Xue Gang.

**Funding acquisition:** Jingjing Luo.

**Investigation:** Jingjing Luo.

**Methodology:** Jingjing Luo, Feifei Wang, Chengting Rong.

**Project administration:**

**Resources:** Jingjing Luo.

**Validation:** Jingjing Luo.

**Visualization:** Jingjing Luo, Nana Zhang.

**Writing – original draft:** Jingjing Luo.

**Writing – review & editing:** Jingjing Luo, Xinan Wu, Xue Gang.
